# Expertise of European Clinical Trial Units in Conducting and Managing Cross-Border Pediatric Clinical Trials for Rare Diseases

**DOI:** 10.1007/s43441-026-00997-x

**Published:** 2026-06-18

**Authors:** Begonya Nafria, Segolene Gaillard, Martine Dehlinger-Kremer, Pamela Dicks, Ricardo M. Fernandes, Pirkko Lepola, Gunter F. Egger, Lucy Bray, Barbara E. Bierer, Bob Phillips

**Affiliations:** 1 Patient Engagement in Research Department, Institut de Recerca Sant Joan de Déu (IRSJD), Santa Rosa 39-57, 08950 Esplugues de Llobregat, Spain; 2 Universitat Internacional de Catalunya, Immaculada, 22, 08017 Barcelona, Spain; 3 European Young Person’s Advisory Groups Network – eYPAGnet, Barcelona, Spain; 4 Centre d’Investigation Clinique, Hospices Civils de Lyon, Lyon, France; 5 UCB Biosciences, Brussels, Belgium; 6 NHS-NRS Children, NHS Grampian, Royal Aberdeen Children’s Hospital, Aberdeen, UK; 7 Conect4children-Stichting, Utrecht, The Netherlands; 8 Faculdade de Medicina, Lisbon, Portugal; 9 Helsinki University Hospital and University of Helsinki, Helsinki, Finland; 10 European Medicines Agency (EMA), Amsterdam, The Netherlands; 11 European Network of Paediatric Research at the European Medicines Agency (Enpr-EMA), European Medicines Agency (EMA), Amsterdam, The Netherlands; 12 Edge Hill University, Ormskirk, UK; 13 Multi-Regional Clinical Trials Center of Brigham and Women’s Hospital and Harvard, Boston, MA USA; 14 Division of Global Health Equity, Department of Medicine, Brigham and Women’s Hospital, Boston, MA USA; 15 Department of Medicine, Harvard Medical School, Boston, MA USA; 16 Candlelighters’ Supportive Care Research Centre, Centre for Reviews and Dissemination, University of York, York, UK

**Keywords:** Cross-border clinical trials, Clinical trial units, Language accommodation, Informed consent process, PROMs, Quality of life scales, Rare diseases, Pediatric

## Abstract

**Introduction:**

Conducting pediatric clinical trials across borders in Europe presents unique challenges, especially when studies target rare conditions, require specialized expertise at participating sites, and demand active family involvement in the research process. Including international patients in clinical trials is essential for both scientific development and equity in healthcare, but it requires adapting study protocols, informed consent processes, and patient-reported outcome measures (PROMs) to ensure trial feasibility in diverse linguistic and cultural contexts. The current regulatory and legislative frameworks offer limited solutions to address the language barriers and logistical complexities that still limit cross-border access to clinical trials. The aim of this study was to investigate the experience, expertise, and practices of managing international participants in the pediatric clinical trials conducted by clinical trial units (CTUs) in 17 European countries.

**Methods:**

To assess experiences with cross-border pediatric clinical trials for rare diseases across multiple domains, three online surveys were developed and disseminated to Clinical Trial Units (CTUs) within healthcare organizations across Europe. The first survey captured information on general expertise, the second focused on reported good practices in trials involving international patients, and the third aimed to identify documented cases of discrimination, particularly those related to native language requirements. All surveys were reviewed by an expert advisory board and distributed through multiple European research networks and other channels. Data were analyzed using descriptive statistics and thematic analysis.

**Results:**

A total of 43 CTUs from 17 European countries participated in this study, representing a mix of children’s hospitals, general hospitals, and university hospitals. International patients who travelled to the 43 CTUs to participate in clinical trials came from 81 different European and non-European countries, mostly from Morocco, Ukraine, Ecuador, and Venezuela.

Among the reported good practices supporting cross-border access (N = 113), the most common involved providing written and verbal translations of informed consent documents, patient-reported outcome measures (PROMs), and quality of life (QoL) scales when these were not available in patients’ native languages. Twenty cases of discrimination were reported, primarily due to language requirements included in eligibility criteria, which negatively affected trial access for non-native speakers. Most of these cases occurred in industry-sponsored trials for rare diseases (65%, n = 13), which imposed significant logistical burdens, including frequent visits and overnight stays at the hospitals conducting the trial.

**Conclusions:**

While some progress has been made in enabling cross-border access to pediatric clinical trials in Europe, substantial barriers remain, particularly regarding native language diversity and support services for international patients and their families. Systemic changes are needed, including routine translations of study materials, professional interpretation, adaptation of PROMs, and the establishment of dedicated support structures. Collaboration across Europe will be necessary to harmonize language requirements when scientifically justified in the eligibility criteria, promote decentralized trial models, and ensure equitable access to clinical trials for all children, regardless of native language or nationality.

**Supplementary Information:**

The online version contains supplementary material available at https://doi.org/10.1007/s43441-026-00997-x.

## Introduction

Conducting and managing pediatric clinical trials entails unique challenges [1–3] due to the specific needs of children and young people compared with other population subgroups. In pediatric studies, the entire family is often involved in participating in the clinical trial [4], which can directly impact parents’ employment [5], the child’s school attendance [6, 7], and the child’s broader psychosocial well-being [8, 9]. These elements must be carefully considered when designing clinical trials for the pediatric population. Patients and caregivers who are involved in protocol design [10, 11] are best positioned to anticipate the burdens and challenges that participants may face during a clinical trial.

In Europe, the Paediatric Regulation (1901/2006) [12] requires the agreement of a Paediatric Investigation Plan (PIP) as part of every new marketing authorization application, new indication or new pharmaceutical form for medicines intended for use in children. The regulation ensures that necessary data are obtained through studies involving children or, through complementary methods such as extrapolation and modeling and simulation approaches [13]. In the case of protocols originally designed for adult development, unless a waiver is granted, they can be adapted as appropriate to the intended pediatric subpopulation taking into account the needs, preferences, and expectations of children and young people. Consequently, recruitment strategies, the informed consent process, patient information materials, patient-reported outcome measures, and other elements must be adapted to the intended participants’ age, maturity, and therapeutic area [14, 15].

Considering that most health conditions affecting children are classified as rare diseases [16, 17], the expertise of health care institutions and clinical trial professionals is essential to ensure the quality of pediatric clinical research. In Europe rare diseases are conditions that affect fewer than 5 people per 10,000 in the European Union) [18]. These studies are typically conducted in highly specialized centers, also known as centers of excellence, which, in Europe, are part of the European Reference Networks (ERNs) [19], promoted by the European Commission. ERNs are virtual networks that connect specialist healthcare providers across Europe to facilitate cross-border knowledge exchange and expertise, crucial for rare diseases where a patient’s local medical environment may not have the necessary specialized knowledge. There are 24 different ERNs targeting rare, low-prevalence, and complex diseases and conditions (pediatric and non-pediatric) that require highly specialized healthcare. Launched in March 2017, the ERNs include 956 specialized healthcare units in 313 hospitals across 26 countries (25 EU Member States plus the EEA country Norway) [20].

Pediatric rare disease clinical trials require specialized expertise and are often multinational due to the limited number of patients in each country [21]. Cross-border access to these studies provides benefits not only to patients who would otherwise lack such opportunities in their country of residence but also to the scientific community by facilitating recruitment and accelerating timelines to trial completion. Beyond the required expertise in pediatric research, the inclusion of international patients in such studies also demands specific resources and competencies from the professionals involved, both within clinical trial units and across the healthcare centers that transfer patients to the sites conducting the studies.

Little is known, however, about the practice and conduct of international cross-border pediatric rare disease clinical trials in Europe. We therefore created three surveys to investigate these trial practices. The first one aimed to collect data about the general expertise of clinical trials units. The second one was designed to gather insight into the reported good practices of trials that included international patients and the third one addressed cases of discrimination against patient access to cross-border clinical trials. The collective data enabled us to map experiences across Europe, identify lessons learned through the analysis of existing practices, and explore factors that prevented cross-border transfers, including potential cases of discriminatory access.

This research was conducted as part of a broader European initiative led by a working group within the European Network of Paediatric Research at the European Medicines Agency (EnprEMA) [22]. The mission of this working group was to evaluate the current landscape of multi-regional and cross-border pediatric clinical trials in Europe with a particular focus on language-related barriers and potential discrimination.

## Materials and Methods

### General Design

Three online surveys were designed to collect data from European Clinical Trials Units (CTUs), most of them members of different ERNs and EnprEMA, regarding their experience in conducting pediatric clinical studies on rare diseases that included international pediatric patients. First, a general questionnaire gathered information on the type of site (public or private), the proportion of commercial versus academic pediatric trials conducted per CTU, the number of international patients enrolled and their countries of origin (European and non-European), the therapeutic area addressed by the studies, and the list of research networks to which the site belonged (Online Appendix 1).

The other two surveys were designed to collect specific data on studies involving international patients (Online Appendix 2) and those in which it was not feasible to include pediatric patients traveling from other countries (Online Appendix 3). Both surveys included trial identification details, such as study type (academic or industry-sponsored), therapeutic indication, patient age, number of patients screened, consent process, patient-facing materials, and the accommodation of PROMs to the patient’s native language. For the survey reporting the exclusion of patients, specific information was collected regarding the reason for exclusion and whether it was due to the patient’s native language or country of residence.

The surveys were administered in English, were designed with the Qualtrics software and accessible on-line.

### Survey Review Drafting Process

The three surveys were designed by members of the European Network of Paediatric Research at the European Medicines Agency (EnprEMA) Working Group on Cross-border Pediatric Clinical Trials. A dedicated external Advisory Board, composed of seven experts in pediatric clinical research along with the Co-chairs of EnprEMA, reviewed the draft surveys and provided suggestions for improvement. The final versions of the three questionnaires underwent a second round of reviews by the EnprEMA Working Group members and again by the Co-chairs of EnprEMA.

### Dissemination

Different methods were used to disseminate the surveys broadly. The surveys were sent by email by different organizations to their memberships: (1) EnprEMA targeting 54 European pediatric research networks, (2) Conect4children, the pan-European Clinical Trials Network, addressed to the twenty Coordinating Hubs of the National Pediatric Clinical Trials Networks across Europe, (3) European Children’s Hospitals Organization (ECHO) to their 13 members, (4) European Reference Networks (ERNs) dissemination through their Coordinating Office, and (5) Innovative Therapies for Children with Cancer (ITCC) to the site members of their consortium. Two reminders were sent. In addition, fourteen webinars and one-to-one meetings were organized to present the goal of this research to the professionals working in clinical trials units conducting pediatric studies across Europe. They were organized from the 10th of May to the 13th of November of 2023. A total of 34 professionals working in CTUs were involved. The dissemination period ran from May 2, 2023, to April 7, 2025.

### Ethics Committee Approval

All survey responses were anonymous, and no personal data was collected (except for the postal code). Each site generated its own code that helped to align the responses from the general survey with the surveys addressed to report practices that permitted and failed to accommodate cross-border participation.

The three surveys, along with the project to which they belong, were approved by the Ethics Committee of the Institut de Recerca Sant Joan de Déu on March 9, 2023 (approval code PIC-40–23). Some sites asked us to provide our Ethics Committee approval of this project in order to secure internal authorization before completing the three surveys.

### Analysis

The data of this research were downloaded from the Qualtrics software as a single.xls file from the three surveys. The principal investigator was responsible for data curation and for conducting the analysis of all completed responses. Statistical analyses were performed using Microsoft Excel (Table 1).

**Table 1:** Key definitions in the context of this research

Good practice	Activities or actions carried out by a sponsor or Clinical Trial Unit (CTU) that facilitate the inclusion of international patients in pediatric clinical trials conducted in a European country different from the patient’s country of residence, specifically addressing any needs related to the patient’s native language In this manuscript, a “*good practice”* specifically refers to an activity or action that can be implemented by various Clinical Trial Units (CTUs), as it can be replicated and scaled up. These activities or actions can be recommended as a model Example: Translating the patient information sheet and informed consent form into English if the family is proficient in this language and the CTU staff can also communicate in English, even though it is not the official language of the site or the native language of the patient’s family. This approach may be necessary when it is difficult to quickly identify a professional translator for the family’s native language
Case of discrimination	Situation in which international patients were excluded from participating in a clinical trial due to their native language or country of residence without any scientific justification, regardless of whether language or country of residence was included in the eligibility criteria for that specific clinical trial. In Europe, the Charter of Fundamental Rights of the European Union^a^ establishes that any discrimination against European citizens based on their native language or country of residence is prohibited

^a^https://www.europarl.europa.eu/charter/pdf/text_en.pdf Accessed: 2nd of February, 2026

## Results

After data curation only the answers fully completed were included in the analysis: (1) 43 responses from the survey aimed at collecting the expertise of clinical trial units conducting pediatric clinical trials in Europe; (2) 113 clinical trials reported as good practices with the participation of international patients that included general data from 50 trials reported by a single CTU based in Danmark; and (3) 20 trials reported as cases in which cross-border pediatric participation was not possible. All data were analyzed using Excel statistical tools, which were also used to generate tables and figures.

### Survey 1: Experience of European Clinical Trials Units Conducting Clinical Studies Incorporating International Patients

**Fig. 1:**
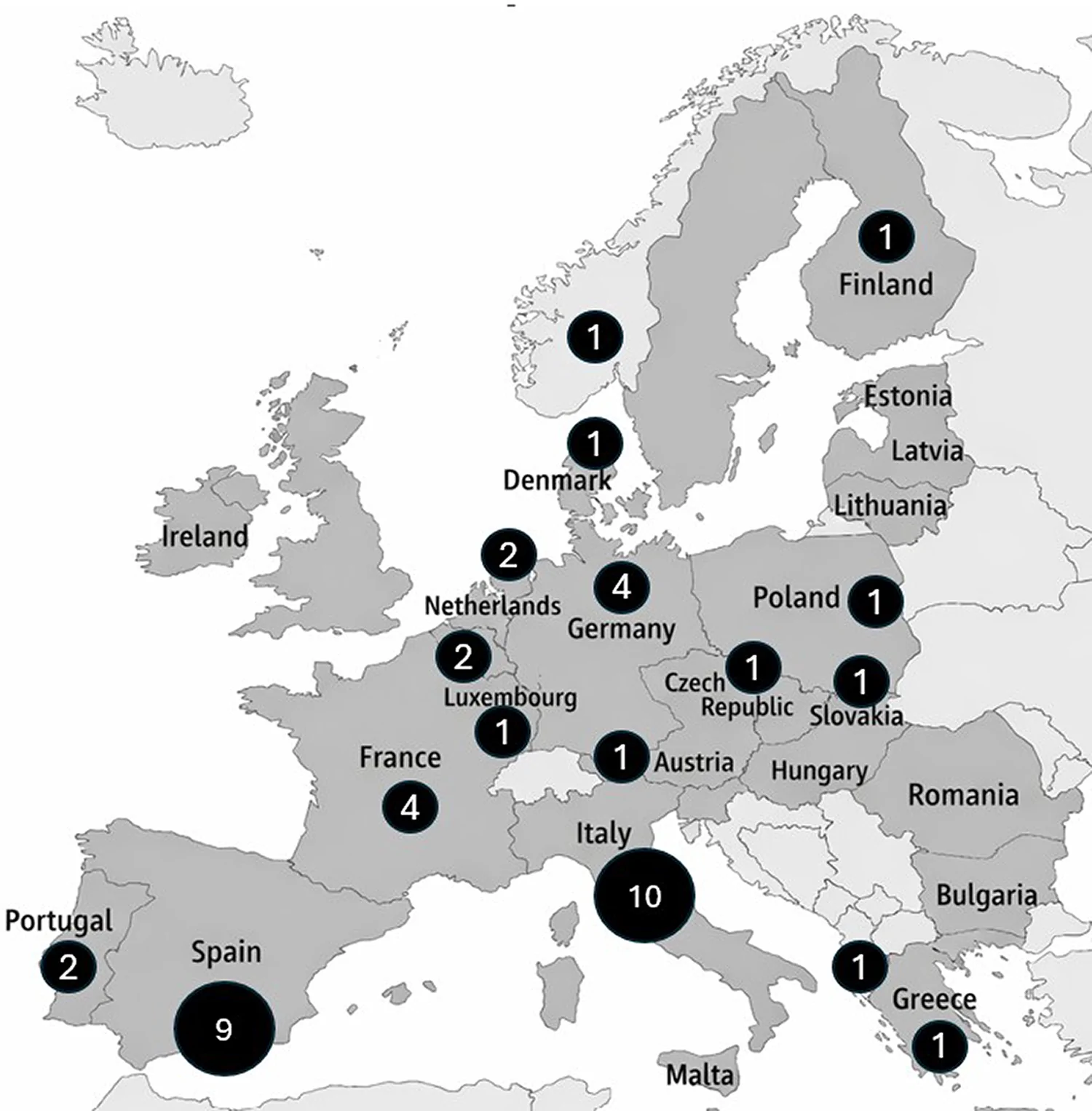
Distribution of the CTUs answers per European countries

After data curation of all three surveys, 43 responses were deemed eligible for analysis, as they provided complete answers to all questions. The participating sites represented 17 different European countries. Only four countries, Italy (n = 10; 23.3%), Spain (n = 9; 20.9%), France (n = 4; 9.3%), and Germany (n = 4; 9.3%) included four or more clinical trial units (Table 2 and Fig. 1).

**Table 2:** Number of clinical trials units per country

Country	N	%
Albania	1	2.3
Austria	1	2.3
Belgium	2	4.7
Czech Republic	1	2.3
Denmark	1	2.3
Finland	1	2.3
France	4	9.3
Germany	4	9.3
Greece	1	2.3
Italy	10	23.3
Luxemburg	1	2.3
Netherlands	2	4.7
Norway	1	2.3
Poland	1	2.3
Portugal	2	4.7
Slovakia	1	2.3
Spain	9	20.9
	43	100.0

The reporting sites were heterogeneous. A total of 32.6% (n = 14) of the clinical trial units were located in children’s hospitals, exclusively focused on the pediatric setting. Another 32.6% (n = 14) were based in general hospitals, while 11.6% (n = 5) were in combined children’s and university hospitals. See Table 3.

**Table 3:** Organizational location of Clinical Trials Units

	N	%
Children’s Hospital	14	32.6
General Hospital (adult and children’s hospital)	14	32.6
Children’s Hospital, University Hospital	5	11.6
University Hospital	3	7.00
General Hospital (adult and children’s hospital), University Hospital	3	7.00
Other (detail)	4	9.3
	43	100.00

The majority of CTUs, 81.4% were part of public health care organizations (n = 35), while 18.6% of the CTUs (n = 8) represented private institutions. The level of experience, measured by the number of clinical trials managed by the CTUs between 2017 and 2022, was heterogeneous. During this six-year period, 9.3% of sites (n = 4) conducted more than 150 clinical studies, 11.6% of CTUs (n = 5) conducted between 100 and 150 trials, and 18.6% of CTUs (n = 8) conducted between 50 and 100 studies, while 60.5% (n = 26) conducted less than 50 trials, reflecting the diversity of organizations involved in pediatric clinical trials. Only some CTUs are exclusively focused on conducting pediatric studies.

The survey asked the managers of the responding CTUs about their institution’s experience in referring patients to other countries to participate in clinical trials. Most centers reported no such experience (67.4%; N = 29). Six centers (14%) transferred patients, but only to European countries. Five centers (11.6%) had experience transferring patients to non-European countries. Three sites (7%) did not respond to this question.

The survey queried the CTU experience in receiving patients from other European countries for healthcare services. 51.2% (n = 22) hospitals reported receiving patients from other European countries. In contrast, 16.3% (n = 7) stated did not report receiving international patients. 32.6% (n = 14) CTUs did not respond to this question.

Most CTUs were members of one or more European clinical research networks (n = 34; 79.1%). Nine (20.9%) CTUs reported that they are not part of any European research network.

**Fig. 2:**
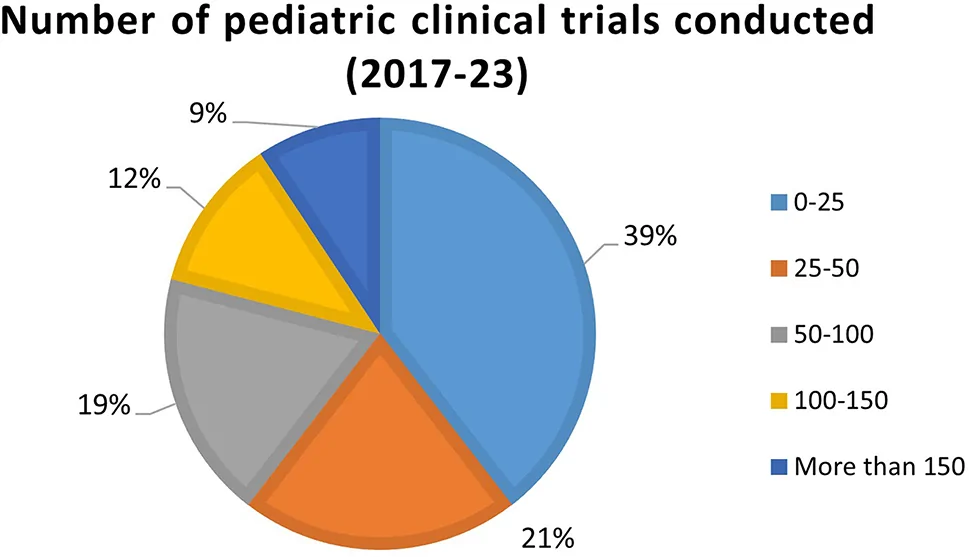
Number of pediatric clinical trials conducted by CTUs, 2017–2023

The survey explored how the CTUs managed international pediatric clinical trial patients, specifically asking whether the organization supported a centralized clinical research unit (one-stop-shop model), one dedicated department or office that coordinated healthcare needs across multiple medical specialties, and/or a department or office to support the participation of international participants in clinical trials (e.g., visa application, translation, etc.). Table 4 summarizes the data collected (Fig. 2).

**Table 4:** Management model of the 43 CTUs analyzed

Centralized clinical research unit (one-stop-shop model)	N	%	Department to coordinate healthcare for rare diseases patients	N	%	Department to provide support to international patients	N	%
Yes	25	58.1	Yes	20	46.5	Yes	10	23.3
No	13	30.2	No	13	30.2	No	21	48.8
Not answered	5	11.6	Not sure	10	23.3	Not sure	12	27.9
		100.00			100.00			100.00

Thirty-nine (90.7%) CTUs provided information about the distribution of industry-sponsored or investigator-led/publicly funded trials as a proportion of overall research activity. In 23 (58.9%) CTUs, industry-sponsored studies accounted for 50% or more of the total activity during the analyzed period. In contrast, only nine centers (23.1%) reported that investigator-led/publicly funded trials represented at least 50% or more of the clinical trials conducted. Four sites (10.3%) reported a balanced distribution of clinical trials by sponsor type, while in three CTUs (7.7%), industry-sponsored studies represented the entirety of their research activity (Table 5).

**Table 5:** Distribution of the clinical trials according to the type of sponsor

	n	%
≥ 50% industry sponsored studies	23	58.9
≥ 50% investigator-led/publicly funded trials	9	23.1
50% industry sponsored + 50% investigator-led publicly funded trials	4	10.3
100% industry sponsored studies	3	7.7
Total	39	100.00

**Fig. 3:**
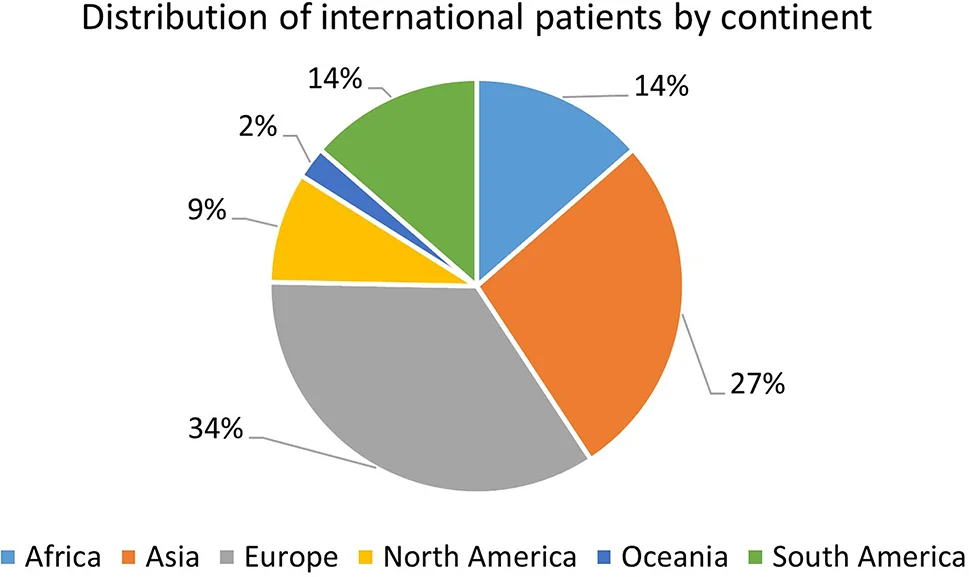
Distribution of international patients by continent of residence

The survey queried the sites that recruited international patients in pediatric clinical trials to report the countries of residence of those patients. A total of 29 sites provided data, reporting that international patients came from 81 different countries, with a total of 176 individual participants from European and non-European countries (see Supplemental Material). Based on the continental distribution of these countries: 28 participants (34.6%) were from Europe, 22 (27.2%) from Asia, 11 (13.6%) from Africa, 11 (13.6%) from South America, seven (8.6%) from North America, and two (2.5%) from Oceania (Fig. 3). Specifically, among the European countries, 17 participants (60.7%) were from Member States of the European Union, while 11 patients (39.3%) were from European countries that are not EU members).

In terms of the countries of residence of international patients participating in clinical trials in Europe, Morocco was the most frequently mentioned, cited by nine sites located in Spain (3), Italy (3), France (2), and Belgium. Ukraine, Ecuador, and Venezuela were each mentioned by six sites, while the United States was mentioned to by five CTUs. Russia, Brazil, Italy, and Peru were each country cited by four sites. Table 6 summarizes the number of European sites that reported international patients. Only one EU Member State (Italy) transferred patients to other Member States, specifically Spain, France, and Austria.

**Table 6:** Countries of residence of international patients participating in a clinical trial in Europe

Patients’ country of origin	Number of European sites that received patients	Continent	Number of clinical trial sites per country	Patient–CTU Language Match (*)
Morocco	9	Africa	Spain (3), France (2), Belgium, Italy (3)	French
Ukraine	6	Europe	Germany, Spain (3), Portugal, Italy	No
Ecuador	6	South America	Spain (4) and Italy (2)	Spanish
Venezuela	6	South America	Spain (4), Italy (2)	Spanish
United States	5	North America	Spain (2), Italy (2), The Netherlands	No
Russia	4	Europe	Spain (2), Germany and Italy	No
Brazil	4	South America	Spain (3), The Netherlands	No
Italy	4	Europe	Spain (2), France, Austria	No
Peru	4	South America	Spain (2), Italy (2)	Spanish

This item analyzes whether the native languages of the transferred patients coincided with the official language of the country where the study was conducted

Only three countries transferred patients to a European country with the same official language. This is the case of Ecuador, Venezuela and Peru which only transferred patients to Spain. In all four countries the official language is Spanish. Language accommodations were provided for families from Morocco (despite French being widely spoken there, it is not an official language), Ukraine, the United States, Russia, Brazil, and Italy.

The fact that Ukraine was the second most frequently mentioned country for patient transfers to other European countries should be analyzed in light of the ongoing war during the data collection period. In this context, some sponsors chose to transfer patients already enrolled in pediatric clinical trials to other European countries, particularly for trials involving life-threatening conditions.

See the Supplemental Material section, where a table summarizes data reported by 26 sites regarding the countries of residence of international patients enrolled in pediatric clinical trials managed by these sites. The information is organized according to the country of the site, the type of organization to which the site belongs, and the continent of residence of the patients.

### Survey 2: Good Practices Reported by the Clinical Trials Units

Fourteen different sites reported on the practices of including international patients in a total of 113 clinical studies. All the good practices were validated by the authors of this manuscript based on the definition of Table 1. One site in Denmark provided the most data, summarizing 50 (44.2%) pediatric phase I and phase II clinical trials of childhood cancer. Spanish sites (n = 9) reported on 36 studies (31.9%), followed by Italian sites (n = 10) with 21 studies (18.5%). The remaining sites (based in Belgium, the Czech Republic, Germany, Greece, Poland, and Sweden) reported on their experience with a single clinical trial. It should be noted to mention that two sites reported on the same oncology trial, and three sites reported on a single allergy study.

The majority of studies (n = 106; 92.9%) pertained to treatment of rare diseases, while seven studies (7.1%) did not. By therapeutic area, the majority of studies were in oncology (n = 86, 76.1%), followed by neurology (n = 16, 14.2%), and the remainder (ophthalmology, infectious diseases, cardiology, allergy, endocrinology, rheumatology, and hematology) each comprised one or two studies, collectively representing 9.7% of the total. Specifically, among the clinical trials addressing childhood cancer conditions, and considering that for the 50 studies reported by the Denmark-based CTU information on the specific disease was provided only under the broad category of “cancer”, leukaemia was the condition accounting for the highest number of reported studies (n = 16; 14.2%).

With respect to clinical trials addressing childhood neurological conditions, Duchenne muscular dystrophy was the disease associated with the highest number of studies (n = 6; 5.3%).

The majority of studies were Phase I, I/II, or II (n = 44, 69.8%), Phase III (n = 18, 28.6%) and Phase IV (n = 1, 1.6%). The status of the studies was balanced across ongoing and/or active, completed, and recruiting. The study duration varied, ranging from 0.5 months to 120 weeks. Of 43 reporting studies, 21 studies (48.8%) required patients to spend at least one night in the hospital, while the remaining 22 studies (51.2%) did not require an overnight stay. In five studies (23.8%), the protocol required patients to stay one night per visit, while in four studies (19%), two nights per visit were required. The maximum number of overnight stays required per visit, according to the clinical trial protocol, was 10 nights in two studies (9.5%). See Supplemental Data and Fig. 3.

A total of 721 patients from European and non-European countries were screened for the 63 studies that reported this data. Of these, 223 patients (30.9%) resided in a European country defined as all countries on the European continent and not limited to EU Member States.

In 31 out of the 63 studies (49.2%), information on translation of the consent and assent process was provided. In 14 studies (45.2%), a verbal translation was provided, either by a professional translator (n = 11, 78.6%) or a community or family member (n = 3, 21.4%), and the documents were signed in the official language of the site. In 17 studies (54.8%), a written translation of the documents into the patient’s native language was provided after submission to the Ethics Committee.

In 21 out of the 63 studies (33.3%), the use of PROMs and QoL scales was reported. In only 9 of these studies (42.8%), the tools for patient/caregiver-reported data were available in a validated version in the native language of the family participating in the clinical trial.

In 33 out of the 63 studies (52.4%), informative resources were offered to patients or caregivers, such as information booklets, electronic patient diaries, multimedia resources, or study websites. In 9 studies (27.3%), no translation was necessary because the patient/family understood the official language of the site. In 24 out of the 33 studies (72.7%), translation into the patient’s/family’s native language was provided using different methods: in five studies (20.8%), translation was provided by the sponsor; in 13 studies (54.2%), written translation was provided by the site. In six studies (25%), verbal translation was provided specifically in English (Tables 7 and 8).

**Table 7:** Number of studies reported by country

Country of the site	Number of studies	%
Denmark	50	44.2
Spain	36	31.9
Italy	21	18.5
Belgium	1	0.9
Czech Republic	1	0.9
Germany	1	0.9
Greece	1	0.9
Poland	1	0.9
Sweeden	1	0.9
Total	113	100.00

**Table 8:** Summary of the reported trial good practices

Good practices:	
Support of logistics by an International Patients Department: VISA, accommodation, etc Verbal translation of the informed consent form by a professional translator, community member, or family member, followed by signature on the official language version of the clinical trial unit form Written translation of the informed consent form into the patient/family’s native language, with signature after submission to the Ethics Committee Complementary informative resources (study guide, booklets, patient diary, etc.) translated into the native language of the patient/family	

### Studies Unable to Accommodate International Patients: Cases of Discrimination

Twenty cases in which international patients could not be accommodated were reported by CTUs from four different countries: Italy (12), Spain (6), the Czech Republic (1), and Sweden (1). 35% of these studies (N = 7) included children (2 to 11 years old) and 5 (25%) children and adolescents (2 to 17 years old). Two studies (10%) included adolescent patients (12 to 17 years old), and the remainder were intended for pre-term, term, infants and toddlers. All the cases of discrimination were validated by the authors of this manuscript based on the definition of Table 1. See Table 9.

**Table 9:** Distribution of cases of discrimination by age of the patients

Patients age	N	%
Children (2 to 11 years old)	7	35
Children (2 to 11 years old), adolescents (12 to 17 years old)	5	25
Adolescents (12 to 17 years old)	2	10
Infants and toddlers (28 days to 23 months)	1	5
Pre-term, infants and toddlers (28 days to 23 months)	1	5
Term newborns (birth to 27 days)	1	5
Term newborns (to 27 days), infants and toddlers (28 days to 23 months)	1	5
Term newborns (to 27 days), infants and toddlers (28 days to 23 months), Children (2 to 11 years old)	1	5
Term newborns (to 27 days), infants and toddlers (28 days to 23 months), Children (2 to 11 years), adolescents (12 to 17 years)	1	5
Total	20	100

**Fig. 4:**
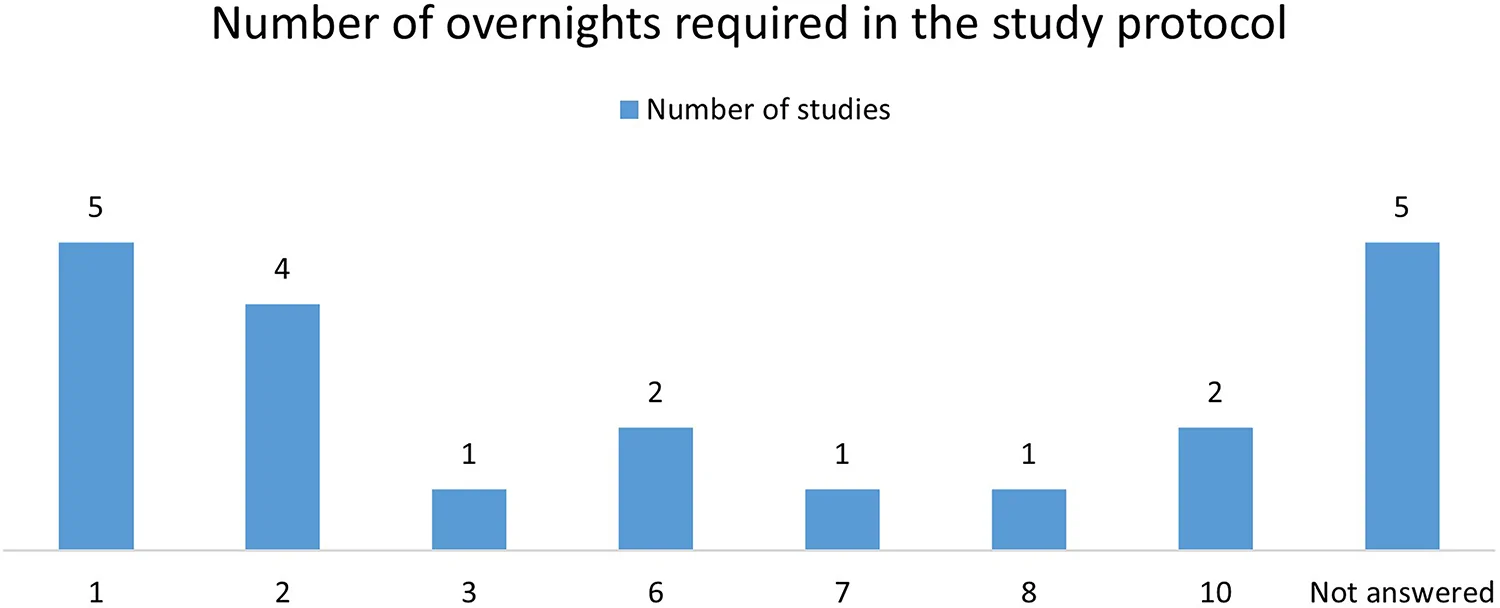
Distribution of studies according to the required number of overnights in the study protocol

Ten out of the 20 studies (50%) were Phase III clinical trials. Five studies were Phase II (25%), four studies Phase I-II (20%), one study was Phase II–III (5%), and none were Phase I only studies (Fig. 4).

**Fig. 5:**
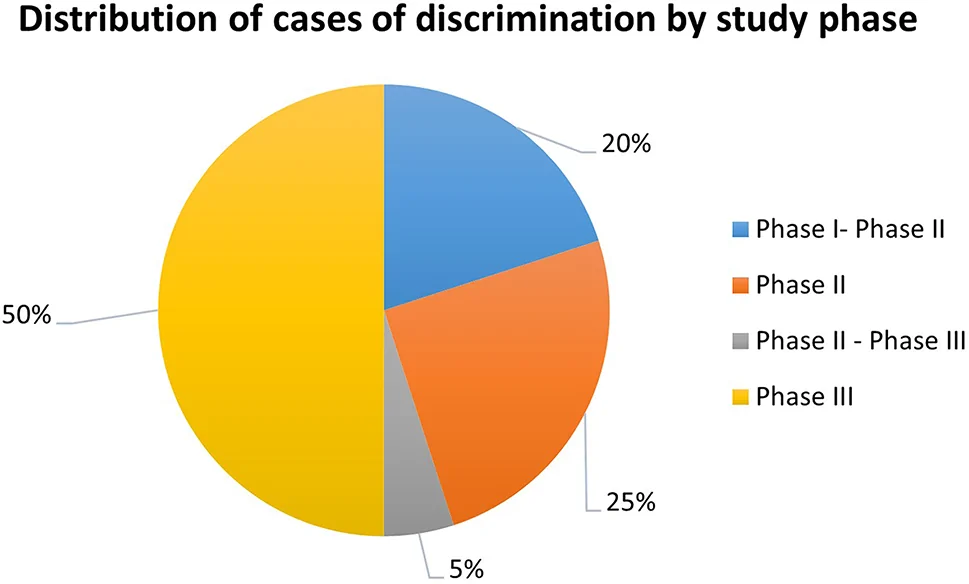
Distribution of study phases

14 (70%) studies were industry-sponsored, and six (20%) were investigator-led/publicly funded trials. At the time of data collection, 8 (40%) studies were completed, 4 (20%) were actively recruiting, 3 (15%) were active but not recruiting, 3 (15%) were suspended, and 2 (10%) were terminated (Table 10). 14 of these studies (70%) addressed treatments for rare pediatric diseases (Fig. 5).

**Table 10:** Distribution of cases of discrimination by study status

Status of the clinical trial	N	%
Completed	8	40
Recruiting	4	20
Active and not recruiting	3	15
Suspended	3	15
Terminated	2	10
Totals	20	100.00

We explored other features of these studies, including their duration (in months), frequency of visits per year, and overall number of visits per trial. The data analyzed were highly heterogeneous: some trials had short durations and few visits, resulting in minimal burden on families, while others had long durations and frequent visits, creating a significant burden and impact on the patient’s and family’s life. Detailed data are available in the Supplemental Files section (Table 11).

**Table 11:** Statistical parameters (Minimum and Maximum value, and Standard Deviation)

	Duration (in months)	Frequency of visits (per year)	Overall number of visits (per clinical trial)
Min	2.5	1	4
Max	128	45	114
Standard Deviation	31.56	9.47	27.57

**Fig. 6:**
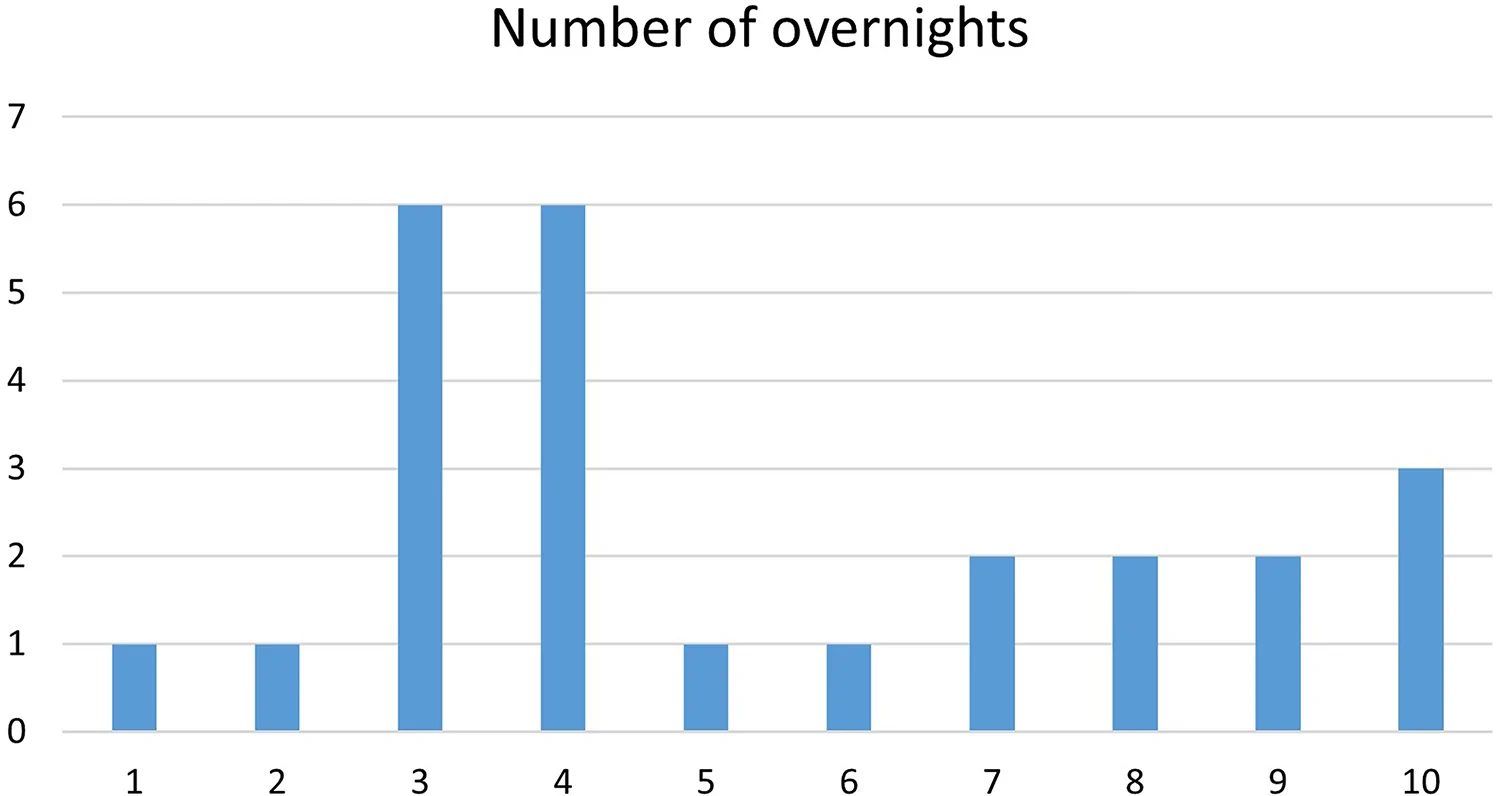
Number of overnights required in the study protocol per medical visit

Ten out of the 20 studies (50%) required patients to stay overnight at the hospital. The minimum number of overnights per visit was one and six the maximum. Figure 6 shows the distribution per trial of the number overnights required to be at the hospital.

**Fig. 7:**
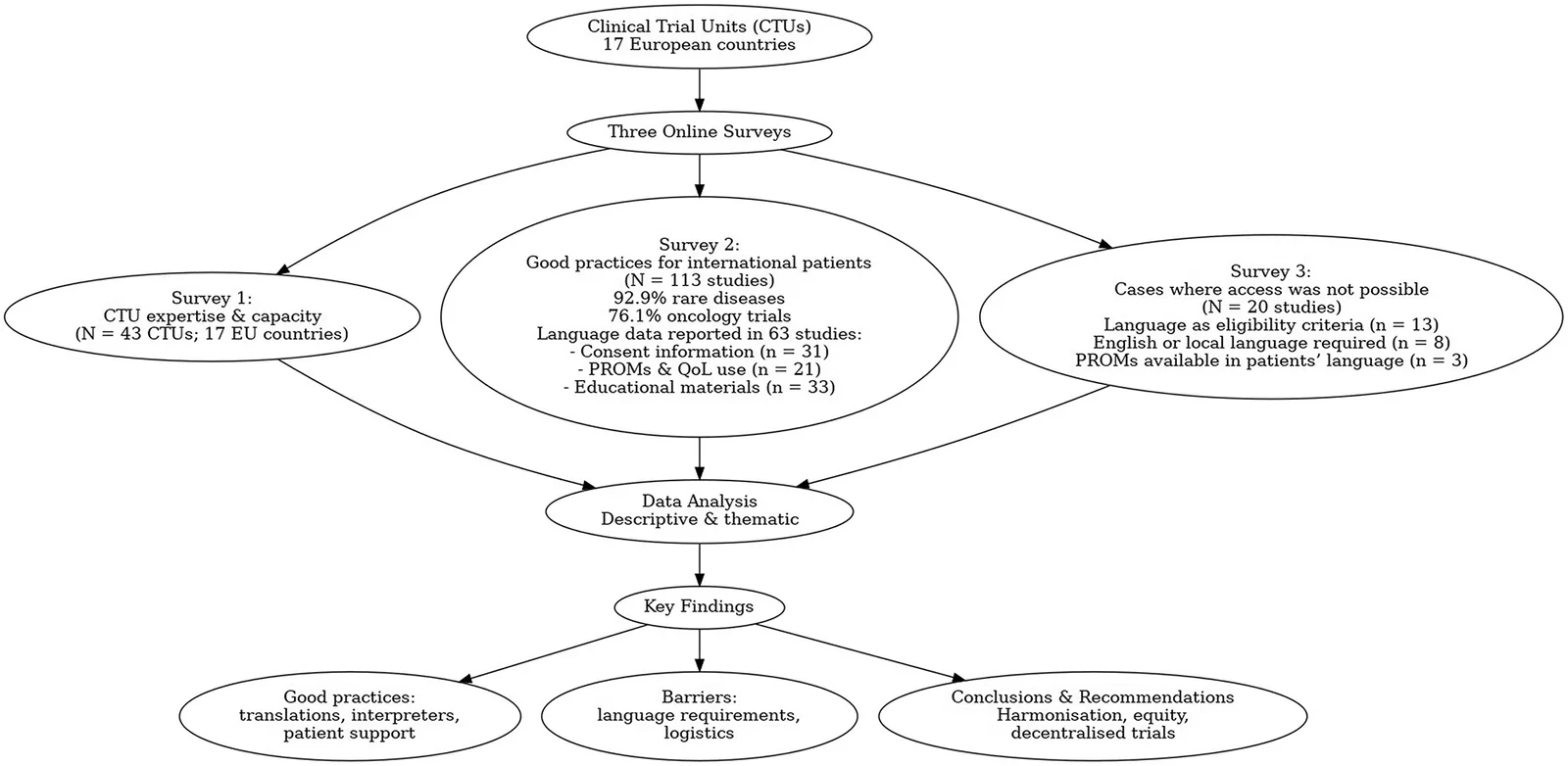
Summary of the data collected with the three online surveys

13 out of the 20 studies (65%) reported information about the consent form in the inclusion criteria. Eight of the 20 studies (40%) included a requirement in the eligibility criteria to speak English or the local language of the site. In three studies (15%), all the PROMs and QoL scales used in the clinical trial were available in the native language of patients who were unable to participate from abroad. In two trials (10%), some of the tools were available in the patient’s native language (Fig. 7).

Professionals at the sites were asked whether they believed that ad hoc verbal translation or professional translation could facilitate patient or family access to completing the PROMs and QoL scales. Two professionals (20%) stated that they did not consider ad hoc verbal translation to be an appropriate method for completing these instruments, while the remaining (n = 8, 80%) considered that linguistic mediation can help address the lack of availability of these tools in the patients’ native language, providing that translation takes into account both language and culture.

The final survey question related to decentralized or hybrid trials and the suitability of accepting patients from abroad in clinical trials that will offer this type of design. Seventeen professionals (85%) considered it beneficial to offer options for decentralization and hybrid clinical trial design. Two professionals (10%) were opposed, and one professional (5%) did not respond to this question.

## Discussion

The results of this study provide an interesting insight into the current landscape, challenges, and opportunities faced by European Clinical Trial Units (CTUs) in managing cross-border pediatric clinical trials. The literature review identified this study as the first conducted in Europe to assess the expertise of CTUs in the conduct and management of pediatric clinical trials for rare diseases that include international patients. Although this may be considered a limitation in terms of sample size, the authors consider that it also represents a strength, as this study provides the first analysis in this specific field that includes the data of the most recognized CTUs conducting pediatric clinical trials across Europe. The data needs to be analyzed in the context of the increasing need for cross-border collaboration, particularly in the context of rare diseases, where patient populations are small and geographically dispersed. The heterogeneity of CTUs who participated in terms of organizational structure, research focus, and experience underscores the complexity of conducting pediatric trials in Europe.

A key finding is the predominance of public institutions and children’s hospitals (70.6%) among the CTUs facilitating cross-border trials, reflecting the specialized expertise required for pediatric research. The authors of this manuscript consider that as an strength the involvement of the most relevant and qualified CTUs from the European pediatric clinical research ecosystem. However, the diversity in the number of trials managed and the varying degrees of integration into European research networks suggest that not all centers are equally equipped to handle the logistical and regulatory complexities of cross-border studies.

The inclusion of international patients in pediatric clinical trials is not only a scientific necessity but also an ethical imperative [23], ensuring equitable access to innovative therapies for children regardless of their country of residence when there is no placebo arm or an open label extension clinical trial [24–26]. The data indicates that while some CTUs have developed effective models for managing healthcare for rare diseases patients (46.5%), including centralized research units and dedicated support departments (58.1%), significant gaps remain. Less than a quarter of CTUs reported having a dedicated department to support international patients (23.3%), indicating a need for broader institutional commitment and resource allocation to provide these services directly through site capabilities.

Language barriers emerged as a critical determinant of access and inclusion. The analysis of reported good practices revealed that while written translation of consent and assent documents into the patient’s native language is increasingly common (54.8%), verbal translation (45.16%), often by professional interpreters, remains a frequent solution. Reliance on ad hoc or community or familial translation can introduce risks related to accuracy and ethical standards. The availability of Patient-Reported Outcome Measures (PROMs) [27] and Quality of Life (QoL) scales [28] in multiple languages remains limited, potentially impacting the validity of patient-centered outcomes and the overall quality of the research.

The reported cases of discrimination, particularly those involving eligibility criteria requiring patients to speak or understand a specific language, underscore the persistence of structural barriers. Exclusion of patients based on language proficiency or requesting to speak a specific native language, rather than clinical or scientific considerations, undermines the principles of equity and scientific rigor. The data collected also suggests that logistical challenges, such as the need for overnight stays and frequent visits, can vary significantly across clinical trials. These challenges can have a profound impact on international families and may contribute to attrition or non-participation.

The findings underscore the importance of harmonizing practices across Europe, not only regarding regulatory requirements, such as using validated PROMs in the patient’s native language, but also in operational aspects, including language accommodation, support services, and patient/family engagement. The experience of CTUs that have successfully included international patients provides valuable lessons for the broader community, highlighting the critical need and benefit of developing a prospective inclusion plan for international participants prior to study initiation. This includes proactive planning, dedicated resource allocation, and collaboration with patient advocacy groups to ensure equitable access and minimize barriers. The authors of this manuscript consider that patients and parents living with a specific health condition targeted by a clinical trial can provide valuable insights when consulted to identify the potential needs of international patients.

Collaboration across Europe will be necessary to harmonize the use of language when scientifically justified in the eligibility criteria, assess decentralized trial models, and implement other necessary changes to ensure equitable access to clinical trials for all children, regardless of native language or nationality.

Beyond the models and expertise of Clinical Trial Units (CTUs), the scientifically and ethically sound design of clinical trials (including a plan for the inclusion of international patients and the involvement of patients and parents in the design) depends largely on funding, which can either facilitate or limit access to cross-border clinical trials. Additional studies are needed to assess the financial impact of including international patients in pediatric clinical trials, as this may enable recruitment according to planned timelines, rather than excluding them and facing delays in recruitment and trial completion. Other aspects that may require further research include those related to the linguistic validation of PROMs and quality-of-life scales when they constitute primary or secondary endpoints in a trial, as well as the need for cultural adaptations, particularly in advanced-phase clinical trials targeting life-threatening conditions.

Finally, the study’s limitations, including the heterogeneity of data, the overall number of answers collected for each survey, potential reporting bias, and the evolving geopolitical context (e.g., the impact of the war in Ukraine) should be acknowledged. Nevertheless, the breadth of responses and the depth of qualitative insights offer a robust foundation for developing practical guidance and policy recommendations.

## Conclusion

This study demonstrates the existence of data good practices; however, while significant progress has been made in enabling cross-border access to pediatric clinical trials in Europe, substantial challenges remain. The expertise and commitment of CTUs are critical to overcoming barriers related to language, logistics, and regulatory complexity. However, the persistence of language-based exclusion and the limited availability of support services for international patients highlight the need for systemic change.

Future research should focus on quantifying the financial implications of including international participants, as doing so may prevent recruitment delays and improve trial completion timelines. Addressing these challenges is fundamental to advancing global collaboration and delivering timely, high-quality care for children in need.

To advance equity and scientific excellence in pediatric clinical research, stakeholders (including sponsors, regulators, CTUs, and patient organizations) must prioritize the development and implementation of standardized good practices. These should include the routine provision of translated study materials, the use of professional interpreters, the adaptation of PROMs and QoL instruments, and the establishment of dedicated support structures for international families.

Future efforts should focus on harmonizing eligibility criteria, assessing the opportunities offered by decentralized and hybrid trial models and fostering cross-border collaboration through European research networks. Most importantly, there should be new funding opportunities especially for academic research groups to enable the conduct of cross-border trials in practice. By addressing these challenges, the European pediatric research community can ensure that all children, regardless of language or nationality, have access to high-quality clinical trials and the potential benefits they offer.

## Supplementary Information

Below is the link to the electronic supplementary material.Supplementary file1 (DOCX 116 kb)

## Data Availability

The full dataset is available upon request.
