# Supplementary material for: Expertise of European Clinical Trial Units in Conducting and Managing Cross-Border Pediatric Clinical Trials for Rare Diseases
Source: Ther Innov Regul Sci. 2026 Jun 18;60(5):1477–89. doi: 10.1007/s43441-026-00997-x (PMC13525932; doi:10.1007/s43441-026-00997-x)
Supplement: Supplementary file 1 [file 43441_2026_997_MOESM1_ESM.docx]

**SUPPLEMENTAL FILES**

| Number of clinical trials that involve patients from 0 up to 17 years old in which the CRU has participated (i.e. recruiting participants) during the period 2017-22 | Sites | % |
| --- | --- | --- |
| 0-25 | 17 | 39,53% |
| 25-50 | 9 | 20,93% |
| 50-100 | 8 | 18,60% |
| 100-150 | 5 | 11,63% |
| More than 150 | 4 | 9,30% |
|  | **43** | **100,00%** |

Summary of data collected by site country, site type, and patients’ country of residence, grouped by continent.

| CTU Country | Type of site | Type of institution | Africa | Asia | Europe | North America | South America | Oceania |
| --- | --- | --- | --- | --- | --- | --- | --- | --- |
|  |  |  |  |  |  |  |  |  |
| Austria | General Hospital | Public |  |  | Italy, Germany, Serbia |  |  |  |
| Belgium | General Hospital | Private | Morocco |  |  |  |  |  |
| Belgium | University Hospital | Public |  | Jordan | United Kingdom |  |  |  |
| Denmark | General Hospital | Public |  |  | Finland, Sweden, Norway, Germany, Cyprus, Poland |  |  |  |
| Finland | General Hospital | Private |  | Kazakhstan | Hungary, Ireland, |  | Honduras |  |
| France | General Hospital, University Hospital | Public | Morocco |  | Italy |  |  |  |
| France | General Hospital | Private | Morocco | Kuwait, South Arabia |  |  |  |  |
| Germany | Children’s Hospital | Public |  | United Arab Emirates, Turkey, Afghanistan, Kirgizstan | Georgia, Ukraine, Byelorussia, Russia, Kosovo |  |  |  |
| Greece | General Hospital | Public |  | Kirgizstan, Kuwait, | Malta |  |  | New Zeeland |
| Italy | Children’s Hospital | Public |  | Armenia, China |  |  |  |  |
| Italy | Children’s Hospital, University Hospital | Public | Senegal, Ivory Coast, Morocco, Egypt, Ghana, Kenya, Kyrgyzstan, Lebanon, Nigeria | Pakistan, Syria, Cambodia, Iran, Iraq | Moldova, Serbia |  | Peru, Venezuela, Argentina, Chile, |  |
| Italy | Children’s Hospital, University Hospital | Private |  |  |  | USA |  |  |
| Italy | General Hospital | Public | Morocco | Siria |  |  | Ecuador, Venezuela, Peru |  |
| Italy | General Hospital | Public | Morocco |  | Ukraine |  |  |  |
| Italy | General Hospital | Private | Ethiopia | Turkey, Japan, Pakistan, Israel, Palestine, | Russia | USA | Bolivia, Guyana, Argentina | Australia |
| Italy | University Hospital | Public | Cameroon |  | Andorra, France | Canada | Ecuador |  |
| Luxemburg | Children’s Hospital | Private |  |  | Belgium, France |  |  |  |
| Netherlands | General Hospital | Public |  | Pakistan, Iraq |  | Aruba, Sint Maarten, USA | Brazil |  |
| Portugal | General Hospital | Public | Rwanda | United Arab Emirates | Romania, Sweden, Switzerland, Ukraine |  | Panama, Uruguay |  |
| Spain | Children’s Hospital | Public | Guinea Equatorial | Philippines | Portugal, Romania, Ukraine, Russia |  | Venezuela, Brazil, Peru, Paraguay, Ecuador |  |
| Spain | Children’s Hospital | Public |  | India | Portugal, Czech Republic, Slovenia, Estonia | USA | Chile, Brazil, Colombia, | Australia |
| Spain | Children’s Hospital | Public | Morocco |  | Italy, The Netherlands |  | Venezuela |  |
| Spain | General Hospital | Public |  |  | Ukraine |  | Paraguay, Ecuador, Venezuela |  |
| Spain | Children’s Hospital, University Hospital | Private | Morocco, Cameroon, Rwanda, | China, Belarus, India, Kazakhstan, Kyrgyzstan, Kuwait, United Arab Emirates, | Poland, Russia, United Kingdom, Germany, Portugal, Czech Republic, Slovenia, Estonia, Italy, The Netherlands, Andorra, France, Hungary, Ireland, Malta, Romania, Sweden, Switzerland, Ukraine, | United States, Canada, | Argentina, Mexico, Peru, Chile, Brazil, Colombia, Venezuela, Ecuador, Honduras, Panama, Uruguay | Australia, New Zealand |
| Spain | General Hospital | Private | Senegal | China |  |  |  |  |
| Spain | General Hospital | Public | Morocco | Turkey, Afghanistan |  | Colombia, Ecuador, Mexico |  |  |

Number of countries and patients transferred to participate in a clinical trial in a EU Member State

| Country | N= | EU Member State | Continent |
| --- | --- | --- | --- |
| Afganistan | 2 | No | Asia |
| Andorra | 2 | No | Europe |
| Arab Emirates | 1 | No | Asia |
| Argentina | 3 | No | South America |
| Armenia | 1 | No | Asia |
| Aruba | 1 | No | North America |
| Australia | 3 | No | Oceania |
| Belgium | 1 | Yes | Europe |
| Bolivia | 1 | No | South America |
| Brazil | 4 | No | South America |
| Byelorussia | 2 | No | Europe |
| Cambodia | 1 | No | Asia |
| Cameroon | 2 | No | Africa |
| Canada | 2 | No | North America |
| Chile | 3 | No | South America |
| China | 3 | No | Asia |
| Colombia | 3 | No | South America |
| Cyprus | 1 | Yes | Europe |
| Czech Republic | 2 | Yes | Europe |
| Ecuador | 6 | No | South America |
| Egypt | 1 | No | Africa |
| Estonia | 2 | Yes | Europe |
| Ethiopia | 1 | No | Africa |
| Finland | 1 | Yes | Europe |
| France | 3 | Yes | Europe |
| Georgia | 1 | No | Europe |
| Germany | 3 | Yes | Europe |
| Ghana | 1 | No | Africa |
| Guinea Equatorial | 1 | No | Africa |
| Guyana | 1 | No | South America |
| Honduras | 2 | No | North America |
| Hungary | 2 | Yes | Europe |
| India | 2 | No | Asia |
| Iran | 1 | No | Asia |
| Iraq | 2 | No | Asia |
| Ireland | 2 | Yes | Europe |
| Israel | 1 | No | Asia |
| Italy | 4 | Yes | Europe |
| Ivory Coast | 1 | No | Africa |
| Japan | 1 | No | Asia |
| Jordania | 1 | No | Asia |
| Kazakhstan | 2 | No | Asia |
| Kenya | 1 | No | Africa |
| Kirgizstan | 3 | No | Asia |
| Kosovo | 1 | No | Europe |
| Kuwait | 3 | No | Asia |
| Lebanon | 1 | No | Asia |
| Malta | 2 | Yes | Europe |
| Mexico | 2 | No | North America |
| Moldova | 1 | No | Europe |
| Morocco | 9 | No | Africa |
| New Zeeland | 2 | No | Oceania |
| Nigeria | 1 | No | Africa |
| Norway | 1 | No | Europe |
| Pakistan | 3 | No | Asia |
| Palestine | 1 | No | Asia |
| Panama | 2 | No | North America |
| Paraguay | 2 | No | South America |
| Peru | 4 | No | South America |
| Philippines | 1 | No | Asia |
| Poland | 2 | Yes | Europe |
| Portugal | 3 | Yes | Europe |
| Romania | 3 | Yes | Europe |
| Russia | 4 | No | Europe |
| Rwanda | 2 | No | Africa |
| Senegal | 2 | No | Africa |
| Serbia | 2 | No | Europe |
| Sint Maarten | 1 | No | North America |
| Siria | 1 | No | Asia |
| Slovenia | 2 | Yes | Europe |
| Sweden | 3 | Yes | Europe |
| Switzerland | 2 | No | Europe |
| Syria | 1 | No | Asia |
| The Netherlands | 2 | Yes | Europe |
| Turkey | 3 | No | Asia |
| Ukraine | 6 | No | Europe |
| United Arab Emirates | 2 | No | Asia |
| United Kingdom | 2 | No | Europe |
| United States | 5 | No | North America |
| Uruguay | 2 | No | South America |
| Venezuela | 6 | No | South America |
|  | 176 |  |  |

Status of the clinical trials reported as good practices

| Status of the trial | Number of studies | % |
| --- | --- | --- |
| Ongoing | 16 | 25.40% |
| Completed | 15 | 23.81% |
| Recruiting | 15 | 23.81% |
| Active and not recruiting | 11 | 17.46% |
| Terminated | 4 | 6.35% |
| Active and not recruiting | 1 | 1.59% |
| Temporarily Halted | 1 | 1.59% |
|  | 63 | 100.00% |

**Appendix 1**

**QUESTIONNAIRE ADDRESSED TO THE CLINICAL RESEARCH UNIT MANAGER, DIRECTOR OR COORDINATOR**

Thank you for your interest in participating in this research initiative promoted and approved by Enpr-EMA.

Your contribution is important to analyse whether language or other differences limit access of paediatric patients to interventional, potentially therapeutic, clinical trials not offered at their country of residence.

In this questionnaire, we will ask you about general data from your Clinical Research Unit from 2017 and 2022. **The data refer to paediatric clinical studies**. You can download the questions and provide the data in this questionnaire once you have collected all the information.

Please take into account that this research project is focused in these criteria:

- **Age of the patients**: from 0 to 17 years old
- **All diseases, disorders, conditions or syndromes** affecting by children and young people are eligible in the data collection.
- **Clinical trials focused in medicines, vaccines and medical devices** (from phase I, II, III and IV).
- Not only the patients travelling from abroad to participate in a clinical trial are eligible. **You can report data of patients that live in your country but are not native speakers** (short-term residents, immigrants, refugees, etc.). We ask because language boundaries exist for patients from abroad when they are not fluent in the official language of your country. This research addresses the challenges of language rather than citizenship.
- From patients traveling from abroad to participate in a clinical trial, you can report **patients from European and non-European countries**.
- The term **native language** refers to the language that a person acquires in early childhood is spoken in the family and/or it is the language of the region where the child was born. Also referred in this questionnaire as **mother tongue**.

The team involved in this research appreciate your help!

Clinical Research Unit Code:

**(Please, create a site-specific code of six numbers (e.g. 123456) for your Clinical Research Unit and share this code with the staff of your site that will contribute to this project. It's really important that everyone uses this same code, because the code enables to identify and aggregate the site specific questionnaire data)**

Your role (*):

- Clinical Research Unit Director
- Site coordinator
- Clinical Trial Manager
- Other (detail)…

**Sociodemographic data**

In this section, we are asking to you general data about your site that will be useful for the comparison of the cases of inclusion and exclusion of patients from abroad or with residency is in your country but not competent in the official language.

Country (*):

City (*):

Postal code (*):

Type of site. Select all the options that apply:(*):

- Children’s Hospital
- General Hospital (adult and children’s hospital)
- Primary Health Care Center
- University Hospital
- Other

Type of institution (*):

- Public
- Private

**Clinical trials research activity (2017-2022)**

Now we are going to ask you specifically about the interventional clinical trials executed by your CRU that included paediatric patients (until 17 years old). This refers to clinical trials phase I, phase II, phase III and, phase IV. They can be specifically paediatric clinical trials or adult clinical trials that allowed the participation of adolescents (below 18 years old).

Number of clinical trials that involve patients from 0 up to 17 years old in which the CRU has participated (i.e. recruiting participants) during the period 2017-22 (*):

- 0-25
- 25-50
- 50-100
- 100-150
- More than 150

Which overall percentage of activity represents the commercial and academic paediatric trials at your site?

- Industry sponsored ___ %
- Investigator-led/publicly funded trials _____ %

Number of paediatric clinical trials that the site has been managing during the period 2017-22 including the ones that have not yet been completed or closed during this period (*):

- 0-25
- 25-50
- 50-100
- 100-150
- More than 150

Number of paediatric patients (approximately) screened in the active clinical trials of the site in the period 2017-22: [open field]

From the data reported before, how many patients (approx.) travelled from other European countries to access the trial at your site (see link to list of countries):

Albania

Andorra

Austria

Belgium

Bosnia & Herzegowina

Bulgaria

Croatia

Czech Republic

Denmark

Estonia

Finland

France

Germany

Greece

Hungary

Iceland

Ireland

Italy

Kosovo

Latvia

Lithuania

Luxemburg

Malta

Montenegro

Netherlands

North Macedonian

Norway

Poland

Portugal

Romania

Slovakia

Slovenia

Spain

Sweden

Ukraine

United Kingdom

From the data reported before, the number of paediatric patients (approximately) screened for active clinical trials at your site in the period 2017-22 coming from non-European countries:

- If possible, detail the names of the countries of origin:

Have you referred paediatric patients from your site to travel to other countries to participate in a clinical trial?

- Yes, but only to European countries.
- Yes, internationally.
- No, we don’t have experience with this.

Do you know, if your clinical departments receive paediatric patients referred from other European countries to receive healthcare (*)? Yes /No / I don’t know

- If yes, can you ask about how many patients annually (average from 2017-22) are provided care in your clinical departments coming from abroad?

Do you know, if your clinical departments received paediatric patients referred from other non-European countries to receive healthcare (*)? Yes /No / I don’t know

- If yes, how many patients annually (average from 2017-22) are provided care in your clinical departments coming from abroad?

**Medical expertise**

Select all the paediatric subspecialties that apply where regularly the Clinical Research Unit conducted clinical trials during the period 2017-2022:

- Adolescent medicine
- Adolescent Psychiatry
- Allergy and Immunology
- Anaesthesiology
- Bone marrow transplantation
- Behavioural medicine
- Cardiology
- Cardiovascular Surgery
- Child Psychiatry
- Clinical genetics
- Critical care / emergency medicine
- Dentistry and orthodontics
- Dermatology
- Developmental and Behavioural medicine
- Emergency
- Epidemiology
- Endocrinology /diabetes
- Gastroenterology
- General paediatrics
- Gynaecology, obstetrics
- Haematology
- Hepatology
- Infectious diseases
- Intensive care
- Mental Health
- Metabolic diseases
- Neonatology
- Nephrology
- Neurology and neurophysiology
- Neurosurgery
- Nutrition
- Oncology
- Ophthalmology
- Paediatric Orthopaedics
- Otolaryngology
- Pain management
- Palliative care
- Paediatric Rehabilitation
- Public Health
- Primary Paediatrics
- Physical rehabilitation
- Pulmonology and Asthma
- Rheumatology
- Sports medicine
- Sexual abuse and child abuse
- Solid organ transplantation
- Paediatric Surgery
- Urology
- Traumatology
- Vaccinology
- Other (detail)

Is the site a member of one or more European clinical research networks? Yes/No

- (If yes) List of EnprEMA networks (select all that apply): see list at the end of this document

Is your site member of another clinical research network? Please specify:

**Features of the site and the organization**

The clinical trials in your institution are managed by a centralized clinical research unit (*one-stop-shop model*)? Yes/No

Do you have a dedicated department or office in your institution to coordinate the healthcare for rare diseases patients (including paediatric patients) that require the coordination of several medical departments? Yes/No/ Not sure

Do you have a specific department to provide support to international patients participating in clinical trials (VISA process, translation, etc.)? Yes/No

Do you want to share any good practices in terms of inclusion of patients not competent in the official language of your CRU in the studies that you have executed during the period 2017-22?

Do you want to share any issue, limitation or area of improvement to facilitate the inclusion of patients not competent in the official language of your CRU in the studies that you have executed during the period 2017-22?

Thanks for your collaboration!

**Thanks for your interest to participate in the data collection process of the Cross-Border Paediatric Cinical Trials Working Group of Enpr-EMA.

The data you have provided is anonymous and is stored on a secure server at the Hospital Sant Joan de Déu de Barcelona, coordinator of this project.**

Access, rectification or deletion of data, as well as other rights, detailed in the Spanish Organic Law for the Protection of Personal Data (LOPD) and in accordance with Regulation (EU) 2019/679 of the European Parliament and the Council of 27 April 2016 on the protection of people with respect to the processing of personal data as well as the transfer of said data (GDPR), has to be executed through a written communication to the Principal Investigator of this project: Begonya Nafria (begonya.nafria@sjd.es)

**List of Enpr-EMA networks:**

AEPC - (Association for European pediatric and congenital cardiology) Task Force on Clinical (drug) Trials

CEPOETA - Central European Pediatric Oncology Early Trials Alliance

conect4children - Collaborative Network for European Clinical Trials for Children (c4c)

Duke Clinical Research Institute (DCRI)

EAPRASnet - European Academy of Paediatrics Research in Ambulatory Settings Network

EBMT PDWP - European Blood and Marrow Transplantation Paediatric Diseases Working Party

ECAPN - European Child & Adolescent Psychopharmacology Network

ECFS-CTN - European Cystic Fibrosis Society - Clinical Trials Network

EORTC CLG - Children Leukemia Group of the European Organisation for Research and Treatment of Cancer

EPLTN - European Paediatric Liver Transplantation Network

ESDPPP - European Society of Developmental
Paediatric Perinatal Pharmacology

ESPNIC - European Society of Paediatric and Neonatal Intensive Care medicine Research Network

EUNETHYDIS - European Network for Hyperkinetic Disorders

eYPAGnet - European Young Persons Advisory Groups Network

FINPEDMED - Finnish Investigators Network for Pediatric Medicines

FP-MCRN - Family Pediatricians Medicines for Children Research Network)

GNN - German Neonatal Network

HELPNET - Greek national network for paediatric clinical trials

HunPedNet - Hungarian Primary Care Pediatricians’ Clinical Research Network

I-BFM-SG - International BFM Study Group

INFANT - Irish Centre for Fetal and Neonatal Translational Research

Irish Paediatric Clinical Research Network (IPCRN)

ITCC - Innovative Therapies for Children with Cancer

JSWG of PRES - Juvenile Scleroderma Working Group of the Pediatric Rheumatology European Society

Medicines for Children Research Network (MCRN) - Hungary

MICYRN - Maternal Infant Child & Youth Research Network

Multinational Interdisciplinary Working Group for Juvenile Idiopathic Arthritis associated uveitis

JPeDNet - Japan Pediatric Society Drug Development Network

NETSTAP e.V. - Network of National Paediatricians in Germany

Newcastle-CCLG - Newcastle Children's Cancer and Leukaemia Pharmacology Studies Group

NIHR CRN-Children - National Institute for Health Research Clinical Research Network-Children

NorPedMed - Medicines for Children Research Network

OKIDS - Medicines for Children Research Organization Austria

PCIC - Paediatric Clinical Investigation Center, Belgium

PEDDCReN - Paediatric European Digestive Diseases Clinical Research Network

Pedmed-NL - Medicines for Children Research Network Netherlands

PEDSTART - French national paediatric research network

PENTA- ID - Paediatric European Network for the Treatment of AIDS and Infectious Diseases

PIBD-Net - Pediatric Inflammatory Diseases Network

PRINTO - Pediatric Rheumatology International Trials Organisation

RECLIP - Spanish Pediatric Clinical Trials Network

Red SAMID - Spanish National Obstetric and Paediatric Research Network

ReSViNET - Respiratory Syncytial Virus Network

RIPPS - Paediatric Investigation into Health Products Network, France

RITIP - Translational Research Network in Pediatric Infectious Diseases, Spain

ScotCRN - Scottish Children's Research Network

SPACE - Severe Paediatric Asthma Collaborative
in Europe

Stand4Kids - Supporting Pediatrics Trials Portugal

SwissPedNet - Swiss Research Network of Clinical Pediatric Hubs

TEDDY - European Network of Excellence for Paediatric Research

TREAT-NMD - Neuromuscular Network

UKPVG - United Kingdom Paediatric Vaccine Group

**Appendix 2**

**QUESTIONNAIRE ADDRESSED RESEARCHERS AND OTHER PROFESSIONALS INVOLVED IN PAEDIATRIC CLINICAL TRIALS**

**GOOD PRACTICES IN THE PARTICIPATION OF CROSS-BORDER ACCESS PAEDIATRIC CLINICAL TRIALS**

Thank you for your interest in participating in this research initiative promoted and approved by Enpr-EMA. Your contribution is important to provide real data to analyse how the access to paediatric patients in cross-border access is managed in Europe solving the boundaries of language.

In this questionnaire, we will ask you data from your experience including patients from abroad in paediatric clinical studies during the period 2017-2022. See below the criteria to report cases.

To answer this questionnaire you will need to collect data from your working experience in the paediatric clinical trials field. You can download the questions and provide the data in this questionnaire once you have collected all the information.

Please take into account that this research project is focused in these criteria:

- **Age of the patients**: from 0 to 18 years old
- **All diseases, disorders, conditions or syndromes** suffered by children and young people are eligible in the data collection.
- **Clinical trials focused in medicines** (from phase I, II, III and IV).
- Not only the patients travelling from abroad to participate in a clinical trial are eligible. **You can report data of patients that live in your country but are not native speakers** (short term residents, immigrants, refugees, etc.) because they were not born in your country. The language boundaries can be the same of the patients from abroad when they don't speak well the official language of your country. This research addresses the challenges of language rather than citizenship.
- From patients travelling from abroad to participate in a clinical trial, you can report **patients from European and non-European countries**.
- The term **native language** refers to the language that a person acquires in early childhood because it is spoken in the family and/or it is the language of the region where the child was born. Also referred in this questionnaire as **mother tongue**.

**Please, for each clinical trial in which you want to report good practices complete one questionnaire.**

The team involved in this research appreciate your help!

Clinical Research Unit Code:

**Please, add here the 6 digit code of your Clinical Research Unit that has been distributed to you (e.g. 123456). This code needs to be the same in all the questionnaires completed by the staff of your site contributing this project.**

**If you don't have this code, please contact your organization´s Clinical Research Unit Coordinator. The code enables to identify (anonymously) and aggregate the site-specific questionnaire data.**

Country of the site:

Your role:

- Principal investigator
- Site-investigator
- Co-investigator or associate investigator
- Project manager
- Research Nurse or Research assistant
- Other…

**Clinical trial features:**

Complete this section providing information about the clinical trials that you have been involved in as a professional and in which you were able to include children and young people (aged 0 to 18 years old) from abroad during the period from 2017-22. **You can report a maximum of 10 studies.** Please, prioritize the ones in which you included more patients from different European countries in preference to those from non-European countries.

Title of the protocol (be accurate) (*):

If you know, refer one of these identifiers (*):

- Clinicaltrials.gov
- EUDRA CT
- CTIS Identifier

Age of the patients that you recruited (select all that apply) (*):

- Pre-term
- Term newborns (to 27 days)
- Infants and toddlers (28 days to 23 months)
- Children (2 to 11 years old)
- Adolescents (12 to 17 years old)
- Other (detail the range of ages)

Interventional study type (select all that apply i.e. Phase I/II) (*):

- Early phase I
- Phase I
- Phase II
- Phase III
- Phase IV

Type of study (*):

- Industry sponsored
- Investigator-led/publicly funded trials

Current status of the study’s recruitment:

- Recruiting
- Active and not recruiting
- Suspended
- Terminated
- Completed

Disease Name (*):

Rare disease: □ Yes □ No

Study time duration per patient (in months) (*):

Frequency of visits per year (*):

Overall number of visits during the clinical trial: (*)

Did the protocol require that patients stayed overnight at the hospital (out of the home)? Yes/No

- If yes, up to how many nights per visit (average)?

**Patients from abroad involved in the clinical trial** (European and non-European countries)

Total number of patients screened in your site for this trial (from European and non-European countries):

From the previous number, detail the **number of patients screened from European countries** (not including the country of your site):

Countries of origin of the patients (select all the options that apply):

Albania, Andorra, Austria, Belgium, Bosnia and Herzegovina, Bulgaria, Croatia, Republic of Cyprus, Czech Republic, Denmark, Estonia, Finland, France, Germany, Greece, Hungary, Ireland, Iceland, Italy, Kosovo, Latvia, Lichtenstein, Lithuania, Luxembourg, Malta, Montenegro, Netherlands, North Macedonia, Norway, Poland, Portugal, Romania, Slovakia, Slovenia, Spain, Sweden, Switzerland, Ukraine, United kingdom… other (detail)

Mother tongue of the patients screened:

Albanian, Bosnian, Bulgarian, Catalan, Croatian, Czech, Danish, Dutch, English, Estonian, Finnish, French, German, Greek, Hungarian, Icelandic, Irish, Italian, Latvian, Lithuanian, Luxembourgish, Macedonian, Maltese, Norwegian, Polish, Portuguese, Romanian, Serbian, Slovak, Slovenian, Spanish, Swedish, Ukraine, other (detail)

If any mother tongue of the patients screened is not in the previous list, detail here the names:

How many patients/families coming from European countries (approx.) were competent in the language of the country were the study was performed if this was not the mother tongue of the patients/parents?

How many patients/families coming from European countries were competent in English if English this was not the mother tongue of the patients/parents?

What was the final number of patients who came from other European countries and were enrolled in the trial?

Have any patients from abroad dropped out / withdrew from the study due to travel or family inconvenience? Yes/No

**Number of patients screened from non-European countries:**

Countries of residence of the patients (open field):

Mother tongue of the patients recruited (open field):

How many patients/families coming from non-European countries (approx.) were competent in the language of the country were the study was performed if this was not the mother tongue of the patients/parents?

How many patients/families coming from non-European countries were competent in English if this was not the mother tongue of the patients?

Have any patients from abroad dropped out / withdrew from the study due to travel or family inconvenience? Yes/No

Provide any details if possible (open field)

What was the final number of patients who came from non-European countries and were enrolled in the trial?

If you know, how many of these patients meet these criteria:

- European refugees
- European immigrants
- European temporary residents (work or study reasons)
- Non-European refugees
- Non-European immigrants
- Non- European temporary residents (work or study reasons)

**Consent and assent**

Regarding the consent and assent process, please select the options with regard to how the translation process was managed:

- Written translation of the documents into the mother tongue of the patient and submitted to Ethics Committee
- Verbal translation and signature of the document in the official language of the site:

If the translation was verbal, please detail how it was done:

- Translation through a trained interpreter
- Translation through a community or family member who speaks the native language of the patient
- Translation through a digital tool such as Google translator

In the protocol, did the eligibility criteria include the capacity to understand and sign the ICF in the clinical trial? Yes/No/I don’t know

**Patient Reported Outcome Measures (PROMs) and Quality of Live (QoL) scales**

If PROMs and QoL scales were used in this clinical trial, were they provided in the mother tongue of the patients who participated from abroad? Yes/No/ some of them

- If not, can you explain the process that you followed in order to facilitate the patient/family report the information that the clinical study required? (open field)

Can you report the PROMs and QoL that were part of this study (name of the scale or questionnaire)?

**Other information addressed to the patients involved in the clinical trial**

Did the trial offer informative resources to the patients? Yes/No

Which type of resources (select all that apply)?

- Information booklet
- Patient diary
- Electronic patient diary
- Multimedia resources (video, game, etc.)
- Website
- Others

How was access to these resources adapted for the patients coming from abroad?

- Translation of specific resources that are necessary to manage the participation in the clinical study (e.g. patient diary)
- Translation of all the resources addressed to the patients
- These resources were not translated
- Other options (detail):

Can you share “what worked well” when you include patients from abroad in a clinical trial not competent in the official language of the site where the study was executed?

Can you share “what was most difficult” when you include patients from abroad in a clinical trial not competent in the official language of the site where the study was executed?

**Appendix 3**

**QUESTIONNAIRE ADDRESSED RESEARCHERS AND OTHER PROFESSIONALS INVOLVED IN PAEDIATRIC CLINICAL TRIALS**

**CASES OF DISCRIMINATION IN THE PARTICIPATION OF CROSS-BORDER ACCESS PAEDIATRIC CLINICAL TRIALS**

Thank you for your interest in participating in this research initiative promoted and approved by Enpr-EMA. Your contribution is important to provide real data to analyse how the access to paediatric patients in cross-border access is managed in Europe solving the boundaries of language.

In this questionnaire, we will ask you data from cases of excluding patients to participate in clinical trials from abroad during the period 2017-2022. See below the criteria to report cases.

To answer this questionnaire you will need to collect data from your working experience in the paediatric clinical trials field. You can download the questions and provide the data in this questionnaire once you have collected all the information.

Please take into account that this research project is focused in these criteria:

- **Age of the patients**: from 0 to 18 years old
- **All diseases, disorders, conditions or syndromes** suffered by children and young people are eligible in the data collection.
- **Clinical trials focused in medicines** (from phase I, II, III and IV).
- Not only the patients travelling from abroad to participate in a clinical trial are eligible. **You can report data of patients that live in your country but are not native speakers** (short term residents, immigrants, refugees, etc.) because they weren’t not born in your country. The language boundaries can be the same of the patients from abroad when they don't speak well the official language of your country. This research addresses the challenges of language rather than citizenship.
- From patients travelling from abroad to participate in a clinical trial, you can report **patients from European and non-European countries**.
- The term **native language** refers to the language that a person acquires in early childhood because it is spoken in the family and/or it is the language of the region where the child was born. Also referred in this questionnaire as **mother tongue**.

**Please, for each clinical trial in which you want to report cases of discrimination complete one questionnaire.**

The team involved in this research appreciate your help!

Clinical Research Unit Code:

**Please, add here the 6 digit code of your Clinical Research Unit that has been distributed to you (e.g. 123456). This code needs to be the same in all the questionnaires completed by the staff of your site contributing this project.**

**If you don't have this code, please contact your organization´s Clinical Research Unit Coordinator. The code enables to identify (anonymously) and aggregate the site-specific questionnaire data.**

Country of the site:

Your role:

- Principal investigator
- Site-investigator
- Co-investigator or associate investigator
- Project manager
- Research Nurse or Research assistant
- Other…

**Clinical trial features:**

Complete this section providing information about clinical trials in which you have been involved addressing CRU trials that were not able to include children and young people (aged 0 to 17 years old) from abroad during the period from 2017-22. **You can report a maximum of 10 studies.** Please, prioritize the ones in which you couldn’t involve patients from different European countries against the ones from non-European countries.

Title of the protocol (be accurate) (*):

If you know refer one of these identifiers (*):

- Clinicaltrials.gov
- EUDRA CT
- CTIS Identifier

Age of the patients that you recruited (select all that apply) (*):

- Pre-term
- Term newborns (to 27 days)
- Infants and toddlers (28 days to 23 months)
- Children (2 to 11 years old)
- Adolescents (12 to 17 years old)
- Other (detail the range of ages)

Interventional study type (select all that apply i.e. Phase I/II) (*):

- Early phase I
- Phase I
- Phase II
- Phase III
- Phase IV

Type of study (*):

- Industry sponsored
- Investigator-led/publicly funded trials

Current status of the study’s recruitment:

- Recruiting
- Active and not recruiting
- Suspended
- Terminated
- Completed

Disease Name (*):

Rare disease: □ Yes □ No

Study time duration per patient (in months) (*): [Drop down menu with options]

Frequency of the visits per year (*): [Drop down menu with options]

Overall number of visits during the clinical trial: [Drop down menu with options] (*)

Did the protocol require that patients stayed overnight at the hospital (out of home)? Yes/No

- If yes, up to how many nights per visit (average)?

**Patients from abroad not involved in the clinical trial**

Number of screened patients not recruited from European countries:

Albania, Andorra, Austria, Belgium, Bosnia and Herzegovina, Bulgaria, Croatia, Republic of Cyprus, Czech Republic, Denmark, Estonia, Finland, France, Germany, Greece, Hungary, Ireland, Iceland, Italy, Kosovo, Latvia, Lichtstenstein, Lithuania, Luxembourg, Malta, Montenegro, Netherlands, North Macedonia, Norway, Poland, Portugal, Romania, Slovakia, Slovenia, Spain, Sweden, Switzerland, United kingdom, Ukraine… other

If patients from other European countries were excluded to participate in this clinical trial, detail here the names of these countries:

Mother tongue of the screened patients not recruited:

Albanian, Bosnian, Bulgarian, Catalan, Croatian, Czech, Danish, Dutch, English, Estonian, Finnish, French, German, Greek, Hungarian, Icelandic, Irish, Italian, Latvian, Lithuanian, Luxembourgish, Macedonian, Maltese, Norwegian, Polish, Portuguese, Romanian, Servian, Slovak, Slovenian, Spanish, Swedish, Ukraine, other

If patients speaking other European languages were excluded to participate in this clinical trial, detail here the names of these languages:

How many patients/families coming from European countries were competent in English if this was not the mother tongue of the patients?

Number of screened patients not recruited from non-European countries:

Countries of residence of the patients:

Mother tongue of the patients recruited:

How many patients/families coming from non-European countries were competent in English if this was not the mother tongue of the patients or parents?

How many patients/families coming from non- European countries were competent in the language of the country were the study was performed if this was not the mother tongue of the patients/parents?

If you know, how many of the non-eligible **patients coming from non-European countries** screened to this clinical trial **meet these criteria**:

- European refugees
- European immigrants
- European temporary residents (work or study reasons)
- Non-European refugees
- Non-European immigrants Non- European temporary residents (work or study reasons)

Assent and consent

Regarding the assent and consent process:

- In the protocol, did the **eligibility criteria** include the **capacity to understand and sign the ICF** in the clinical trial? Yes/No
- If yes, did the section on consent/assent refer to the local language of the clinical trial site or English as eligibility criteria in the clinical trial protocol?
  - Yes, it referred to local language
  - Yes, it referred to English
  - No, no language requirements were described

**Patient Reported Outcome Measures (PROMs) and Quality of Live (QoL) scales**

Although you are reporting a case of exclusion of a child or young person of participating in a clinical trial, in this section we want to know about the availability of the PROMs and QoL scales of the study in the mother tongue of the patient. The aim of this section is to identify whether the reason for exclusion was the country of residence or the mother tongue of the patient.

Were the PROMs and QoL scales of this clinical trial available in the mother tongue of the patients who were unable to participate from abroad? Yes/No/Some of them

Can you report the PROMs and QoL scales that were part of this study?

From your professional experience, do you think that ad hoc verbal translation or professional translation could be a method to facilitate the access to the patient/family to complete the PROMs and QoL scales? Yes/No

Please, explain your answer.

Detail the reason or reasons of the exclusion of the patient to participate in the clinical trials?

- Mother tongue of the patient/family
- Country of residence
- Logistical challenges (e.g. visa)
- Expenses not covered
- Other (detail)

Were the families informed of the reason that their child could not join the clinical trial?

- Yes/No

Approximately how many patients were not eligible for participation due to:

- Mother language
- Country of residence
- Both reasons

Would you/your site accept the participation of patients from abroad if the trial was decentralized or hybrid?

- No
- Yes, in a decentralized model
- Yes, in a hybrid model
